# Effect of sterile tetracycline ophthalmic ointment as an adjuvant to mechanical debridement on the treatment of peri-implantitis: A randomized controlled clinical trial

**DOI:** 10.34172/japid.2022.009

**Published:** 2022-06-15

**Authors:** Mohammad Taghi Chitsazi, Azin Khorramdel, Mohammad Amin Mesforoush

**Affiliations:** ^1^Department of Periodontics, Faculty of Dentistry, Tabriz University of Medical Science, Tabriz, Iran; ^2^Student Research Committee, Faculty of Dentistry, Tabriz University of Medical Science, Tabriz, Iran

**Keywords:** Bacterial infection, dental implant, mechanical debridement, peri-implantitis, tetracycline

## Abstract

**Background:**

Peri-implantitis is an infectious disease that affects the tissues around dental implants, with clinical signs of inflammation and irreversible loss of supporting bone. This study aimed to compare the effect of sterile topical tetracycline ophthalmic ointment as an adjuvant to mechanical debridement with mechanical debridement alone in the treatment of peri-implantitis.

**Methods:**

In this single-blind randomized clinical trial, 32 patients (16 patients in each group) with peri-implantitis were treated topically using sterile tetracycline ophthalmic ointment. Four clinical parameters, including modified bleeding index (mBI), modified plaque index (mPI), probing depth (PD), and clinical attachment level (CAL), were measured at baseline and at 3- and 6-month follow-up intervals.

**Results:**

PD reduction was statistically significant after 3 and 6 months in the test and control groups (*P*=0.001). Also, mPI and mBI reduction rates were significant in the test and control groups (*P*=0.001) after 3 and 6 months. However, in all the samples in the two groups, the mean of CAL before and after treatment was constant, with no significant difference (*P*>0.05).

**Conclusion:**

Using sterile ocular tetracycline ointment could be an adjunctive treatment in improving and enhancing the therapeutic effects of mechanical debridement in the treatment of peri-implantitis. (IRCT20210909052418N1)

## Introduction

 Peri-implantitis is an infectious disease that affects the tissues around dental implants and presents with clinical signs of inflammation and irreversible loss of supporting bone.^1-3^ Although the survival rate of dental implants is very high in individuals, after five years of implant placement, up to 14.4% of implants develop inflammatory reactions of the tissues around the implant and varying amounts of bone resorption.^4^ Several factors are involved in the development of peri-implantitis, and it is now well established that bacterial biofilm is a serious risk factor for the development of peri-implantitis.^5^ Therefore, the main goal of treatment is to remove the pathological bacteria colonized on the surface of the infected implant.^6^ Non-surgical treatment has shown little clinical effect in the treatment of peri-implantitis.^7^ In contrast, surgical treatment is recommended to improve recovery and facilitate surface contamination access.^8^ However, cleaning the implant surface is still difficult due to the geometry of the peri-implant bone. In particular, ultrasonic scalers are not suitable for cleaning the surfaces of contaminated implants.^9^ Because of the existing problems, several adjuvant cleansing methods have been used in the surgical treatment of peri-implantitis, including saline or chemicals and photodynamic therapy.

 Recent studies have reported that surgical treatment, combined with various adjuvant methods in disinfecting the implant surface, produces better clinical outcomes than surgery alone. However, no method has proven superior.^9,10^ In addition, lasers and airflow devices on infected implants have shown a weak effect, mainly due to inadequate access to the involved bone.^9,11^ Numerous studies have evaluated the benefits of topical antibiotics that allow high drug concentrations to be maintained in peri-implant bone involvement.^12,13^ Many studies have evaluated the effects of antibiotics as a supplement to non-surgical treatment and found positive effects on clinical parameters in terms of a significant reduction in pocket probing depth (PD) and bleeding on probing (BOP).^14^ In dogs, topical debridement with systemic amoxicillin and metronidazole has improved peri-implantitis lesions.^15^ In general, topical medication provides a safe and effective treatment that improves patient acceptance.^16^ Since this disease is limited to the periodontal pocket, topical medication in the periodontal pocket is the best option.^17^ The pocket acts as a natural reservoir, providing easy access to the drug.^18^ Since tetracycline is an antibiotic that has been used effectively in the treatment of periodontitis, we hypothesized that it could affect the plaque index, bleeding index, peri-implant pocket depth, and the distance between the implant shoulder and gingival margin reduction after topical treatment. This study compared the effect of sterile tetracycline ocular ointment topically as a supplement to mechanical debridement with mechanical debridement alone in treating peri-implantitis.

## Methods

 In this single-blind randomized clinical trial (IRCT20210909052418N1), 32 patients with peri-implantitis were treated with mechanical debridement or topical use of sterile tetracycline ocular ointment. Patients >20 years of age with peri-implantitis and at least one implant with PD>6 mm, positive BOP, and radiographic signs of bone loss were included in the study. Patients should not have used antibiotics or mouthwashes for at least six weeks. Also, the patients did not have systemic diseases and had not undergone periodontal treatment in the last six weeks before the study.

 In the first visit, demographic data, including smoking and medical and dental history, were taken, and oral examinations were performed along with a clinical examination for plaque index (PI), gingival index (GI), and BOP. In addition, anterior probing was performed by Williams probe, BOP with probe movement, gingival analysis from the crown to the gingival area, CAL with PD>6 mm, and PA radiographs to confirm bone loss>2 mm.

 Before starting the study, the steps of the study were explained to all the patients, and written consent was obtained. Initially, the affected area was molded, and an acrylic stent was to evaluate the amount of pocket and CAL before and after treatment. All the patients underwent SRP by the ImplacareTM plastic curette (Hu Friedy Co., Chicago, United States) and were given oral hygiene instructions. The selected patients were randomly divided into test and control groups using the flip of a coin. In the test group, 1% sterile tetracycline ocular ointment (Iran Nazho Pharmacy, Tehran, Iran) plus mechanical debridement was used, and in the control group, only mechanical debridement was used.

 After local anesthesia with xylocaine (Sina Daru, Tehran, Iran), isolation, and drying of surfaces, 1% tetracycline ointment (Iran Nazho Pharmacy, Tehran, Iran) was placed in a flexible periodontal pouch with a blunt cannula without traumatizing or damaging the periodontal tissues^19,20^ This treatment process was repeated every 48 hours for two weeks, and at the end of each treatment period, a Coe-Pak bandage (GC Co., Tokyo, Japan) was applied. Then, four clinical parameters, including mBI, mPI, PD, and CAL, were measured at - and 6-month follow-up intervals.

 Student’s t-test, paired-sample t-test, and chi-squared test were used to compare variables between groups and follow-up intervals. The variables mPI, PD, CAL, and mBI were scored from zero to a certain number, and the results were presented as the mean ± standard deviation of the changes. The values ​​of the clinical parameters recorded around each implant are used to obtain the average implant score for each parameter. The normality of the data was assessed with the Kolmogorov-Smirnov test. Statistical analysis was performed using SPSS 17, and statistical significance was set at P<0.05.

## Results

 This clinical trial was conducted to compare the effect of sterile tetracycline ocular ointment with mechanical debridement in treating peri-implantitis in 32 patients. The patient’s demographic data are presented in Table 1.

**Table 1:** Ptients’ demographic data

**Variable**	**Test group**	**Control group**	**P-value**
**Age (years)**	31.81±4.94	32.75±5.07	0.601
**Gender**			0.639
Male	9 (56.3%)	9 (56.3%)	
Female	7 (43.8%)	7 (43.8%)	

Table 2 presents a comparison of patients’ clinical findings, including modified plaque index, modified bleeding index, pocket depth, and clinical attachment level values between the two groups at baseline and follow-up intervals.

**Table 2:** Patients’ clinical parameters in the two groups at baseline and follow-up intervals

**Parameter**	**Test group**	**Control group**	**P-value**
**mPI**			
**Base**	2.31±0.87	2.25±0.85	0.839
**3-month follow-up**	1.12±0.50	1.25±0.57	0.518
**6-month follow-up**	0.87±0.61	0.62±0.50	0.219
**Time (baseline-3 month)**	1.18±0.91	1.00±0.73	
**P-value**	0.001	0.001	
**Time (baseline-6 months)**	1.43±0.89	1.62±0.88	
**P-value**	0.001	0.001	
**Time (3 months-6 months)**	0.25±0.57	0.62±0.61	
**P-value**	0.104	0.001	
**mBI**			
**Base**	0.75±0.68	0.56±0.51	0.387
**3-month follow-up**	0.33±0.21	0.37±0.22	0.705
**6-month follow-up**	0.23±0.40	0.27±0.50	0.253
**Time (baseline-3 months)**	0.42±0.46	0.19±0.29	
**P-value**	0.001	0.001	
**Time (baseline-6 months)**	0.52±0.28	0.29±0.11	
**p-value**	0.002	0.009	
**Time (3 months-6 months)**	0.11±0.19	0.10±0.21	
**P-value**	0.001	0.001	
**PD (mm)**			
**Base**	6.87±0.95	6.56±0.81	0.328
**3-month follow-up**	4.81±0.83	5.18±0.83	0.213
**6-month follow-up**	3.31±0.79	3.93±0.77	0.031
**Time (baseline-3 months)**	2.06±0.92	1.37±1.08	
**P-value**	0.001	0.001	
**Time (baseline-6 months)**	3.56±1.20	2.62±1.02	
**P-value**	0.001	0.001	
**Time (3 months-6 months)**	1.50±0.81	1.25±0.93	
**P-value**	0.001	0.001	
**CAL (mm)**			
**Base**	5.76±0.20	5.48±0.96	0.312
**3-month follow-up**	5.55±0.07	5.49±0.85	0.435
**6-month follow-up**	5.69±0.77	5.46±0.37	0.675
**Time (baseline-3 months)**	0.10±0.01	0.01±0.01	
**P-value**	0.412	0.412	
**Time (baseline-6 months)**	0.07±0.02	0.02±0.01	
**P-value**	0.320	0.320	
**Time (3 months-6 months)**	0.03±0.01	0.01±0.01	
**P-value**	0.212	0.212	

 The results showed that mPI values were not significantly different between the two groups at baseline and 3- and 6-month follow-up intervals (P>0.05). In both groups, mPI values significantly decreased from baseline to the 6-month follow-up interval. A comparison of the mBI values between the two groups at baseline and follow-up intervals showed no statistically significant differences in mBI values between the groups at baseline and 3- and 6-month follow-up intervals (P>0.05). In both groups, mBI values significantly decreased from baseline to 6-month follow-up interval.

 A comparison of pocket depth values between the two groups at baseline and follow-up intervals showed that PD values were not significantly different between the two groups at baseline and 3- and 6-month follow-up intervals (P>0.05). PD values decreased significantly from baseline to the 6-month follow-up interval in both groups. The results showed that the mean CAL in the control group was 5.48±0.96. There was no difference in CAL in the control group before or after treatment. In the test group, the mean CAL was 5.76±0.20. There was no difference in CAL in the test group before and after treatment. In all samples in the two groups, the mean of CAL before and after treatment was constant, with no significant difference (P>0.05).

 The frequency of adverse effects in this study was directly related to the treatment used and the debridement technique. The most common complaint of the patients in the study group was related to the bitter taste of the sterile tetracycline ocular ointment. Another side effect was pain after debridement and headache, which did not affect the treatment process. There were also no cases of acute changes in patients’ vital signs and gingival tissues.

## Discussion

 This clinical trial compared the effect of sterile tetracycline ocular ointment with mechanical debridement in the treatment of peri-implantitis. The pathophysiology of the development and exacerbation of peri-implantitis is related to pathogenic microorganisms. Therefore, the proposed treatment for this condition is a modality that reduces the microbial load, cleans the implant’s surface, and eliminates the inflammation of the mucous tissues around the implant. Although mechanical debridement and oral hygiene are effective factors in reducing the signs and symptoms of inflammation, recent findings have shown that treatment is not sufficient.^21,22^

 In this study, the baseline mPI was 2.31±0.87 in the test group and 2.25±0.85 in the control group, which decreased significantly in both groups at 3- and 6-month follow-up intervals. Significantly, the decline in mPI was 1.18±0.91 in the test group at the 3-month follow-up interval, with 1.00±0.73 in the control group. At the 6-month follow-up interval, the decrease in mPI was 1.43±0.89 in the test group, with 1.62±0.88 in the control group. The decrease in mPI was greater at the 3-month follow-up interval in the test group and more at the 6-month follow-up interval in the control group. Javad et al^23^ reviewed the efficacy of antibiotics in the treatment of peri-implantitis. Consistent with this study, the results showed that where tetracycline was used topically, PI values ​​decreased significantly. The agreement of the results of this study with other studies is probably due to the simultaneous use of antibiotics and mechanical debridement.

 In this study, the baseline mBI was 0.75±0.68 in the test group and 0.56±0.51 in the control group, which decreased significantly at 3- and 6-month follow-up intervals. According to the results, the decrease in mBI in the test group was 0.42±0.46 at the 3-month and 0.52±0.28 at the 6-month follow-up intervals. In contrast, the decreases in mBI in the control group at 3- and 6-month follow-up intervals were 0.19±0.29 and 0.29±0.11, respectively. At both follow-up intervals, the decrease in mBI in the test group was greater than in the control group. In a study by Revent et al^7^ on the topical use of minocycline to treat peri-implantitis with mechanical debridement, postoperative bleeding decreased in the antibiotic group. However, in this study, postoperative bleeding in the antibiotic group evaluated the clinical index of BOP. In a study by Mombelli et al,^13^ consistent with this study, a significant decrease in mBI was observed in the group receiving fibers containing tetracycline, but the rate of decrease was greater than in the present study (i.e., 0.54) at the 6-month follow-up interval. Probably a better reason is the result obtained in the above study due to the use of fibers containing tetracycline and a 10-day dressing in each treatment period.

 In this study, the means of baseline PD in the test and control groups were 6.87±0.95 and 6.56±0.81 mm, respectively, which decreased significantly. Based on the results of this study, the rates of PD decrease in the test group in the 3- and 6-month intervals were 2.06±0.92 and 3.56±1.20 mm, respectively. In contrast, in the control group, the rates of PD reduction at 3- and 6-month follow-up intervals were 1.37±1.08 and 2.62±1.02 mm, respectively. The results show that the decrease in PD was significantly higher in the test group at both follow-up intervals than in the control group. In a study by Mombelli et al^13^ on the treatment of peri-implantitis with topical tetracycline, consistent with this study, in patients in the test group, the PD significantly increased from 0.6 mm to 4.1 mm after treatment and then decreased at 12 months. Probably the reason for the lower decrease in PD in the Mombelli et al^13^study compared with this study was the use of tetracycline as a solution prepared from its capsule; however, in this study, the sterile ocular ointment was used, and a better result was obtained, probably due to the different application protocols of the material. Salvi et al^24^ used chlorhexidine gel and minocycline microspheres to treat peri-implantitis, and consistent with this study, the mean PD ​​declined after 12 months compared with baseline (i.e., 1.6 mm). The reason for the agreement between the current study results and the above study is the use of tetracycline was associated with mechanical debridement compared with the control group (i.e., mechanical debridement). In addition, the rates of PD reduction in this study at 3- and 6-month follow-ups were greater than in the above study; however, in this study, compared to the study by Salvi et al,^24^ more improvement was achieved in a shorter period, probably due to the use of sterile tetracycline ointment, while in the above study, fibers containing tetracycline were used. Toledano et al^25^ reviewed 365 patients with implantitis and 463 implants. After treatment with topical antibiotics, the results showed that PD values ​​of 1.40 mm (1.98–0.82 mm at 95% CI) decreased. In contrast to this study, the rate of PD reduction was lower in the above study. This study is the only study to use sterile tetracycline ointment. According to the available evidence, tetracyclines, due to their beneficial effect on microorganisms involved in peri-implantitis and properties such as better absorption, protein binding, good tissue diffusion, and anticoagulant activity; are a good medication regimen for treating periodontitis.^18^ The reason for the better treatment outcomes in the current study, contrary to the above study, is probably related to the properties of tetracycline compared to other antibiotics. In contrast to this study, Park et al^26^ investigated the effect of tetracycline in treating peri-implantitis, reporting no difference between mechanical debridement alone and supplementing tetracycline with mechanical debridement to improve PD and mBI parameters. The lack of treatment results in the study might be attributed to the small sample size.

 The present study showed that the mean CAL in the control group was 5.48±0.96. There was no difference in CAL in the control group before or after treatment. In the test group, the mean CAL was 5.76±0.20. There was no difference in CAL in the test group before and after treatment. In all the samples in the two groups, the mean CAL before and after treatment was constant, with no significant difference (P>0.05). In a study by Nadig et al,^27^ in contrast to this research, the reduction in CAL six months after treatment with tetracycline-containing fibers was 1.02 mm. Nadig et al^27^ used chlorhexidine chips and tetracycline fibers, which is why the CAL values improved after treatment compared to the current research. Similar to this study, Kennedy et al^28^ compared the efficacy of tetracycline-containing fibers in treating peri-implantitis with mechanical debridement as a baseline treatment with topical tetracycline in combination with systemic antibiotics. The results showed no statistically significant differences between baseline CAL values six months after treatment in patients treated with tetracycline. The lack of improvement in patients treated with tetracycline is probably due to no use of mechanical debridement in these patients.

 The main limitations of this study are small sample size, unicentric sampling, and lack of microbiological profile of the lesion site after treatment, which should be examined in future studies.

## Conclusions

 The present study results showed that using sterile ocular tetracycline ointment could be an adjunctive treatment to improve and enhance the therapeutic effects of mechanical debridement in the treatment of peri-implantitis.

## Authors’ contributions

 AK prepared the proposal, set and entered the results of the studies and their interpretation, prepared the final report and results, and wrote the manuscript. MCT supervised the design and execution of the study and prepared the final report. MAM contributed to the preparation of the proposal, conducted the research, and collected the data. All authors approved the final manuscript.

## Funding

 The work was supported by the Dental and Periodontal Research Center, Tabriz University of Medical Sciences, Tabriz, Iran.

## Availability of data

 The datasets used and/or analyzed during the current study are available from the corresponding author on reasonable request.

## Ethics approval

 The study was approved by the Research Ethics Committee of Tabriz University of Medical Sciences (protocol number: IR.TBZMED.REC.1400.516) and registered with the local World Health Organization Registry Network (IRCT20210909052418N1).

## Competing interests

 The authors declare that they have no competing interests.
